# Hot excited state management for long-lived blue phosphorescent organic light-emitting diodes

**DOI:** 10.1038/ncomms15566

**Published:** 2017-05-31

**Authors:** Jaesang Lee, Changyeong Jeong, Thilini Batagoda, Caleb Coburn, Mark E. Thompson, Stephen R. Forrest

**Affiliations:** 1 Department of Electrical and Computer Engineering, University of Michigan, Ann Arbor, Michigan 48109, USA; 2 Department of Chemistry, University of Southern California, Los Angeles, California 90089, USA; 3 Department of Physics, University of Michigan, Ann Arbor, Michigan 48109, USA; 4 Department of Materials Science and Engineering, University of Michigan, Ann Arbor, Michigan 48109, USA

## Abstract

Since their introduction over 15 years ago, the operational lifetime of blue phosphorescent organic light-emitting diodes (PHOLEDs) has remained insufficient for their practical use in displays and lighting. Their short lifetime results from annihilation between high-energy excited states, producing energetically hot states (>6.0 eV) that lead to molecular dissociation. Here we introduce a strategy to avoid dissociative reactions by including a molecular hot excited state manager within the device emission layer. Hot excited states transfer to the manager and rapidly thermalize before damage is induced on the dopant or host. As a consequence, the managed blue PHOLED attains T80=334±5 h (time to 80% of the 1,000 cd m^−2^ initial luminance) with a chromaticity coordinate of (0.16, 0.31), corresponding to 3.6±0.1 times improvement in a lifetime compared to conventional, unmanaged devices. To our knowledge, this significant improvement results in the longest lifetime for such a blue PHOLED.

## Abstract (web summary)

Large-scale commercialization of organic light-emitting diodes is impeded by the short operational lifetime of blue emitting materials. Lee *et al*. show a strategy to manage the energy dissipation on molecular dissociation using dopants with high triplet exciton energy that improves device stability.

Organic light-emitting diodes (OLEDs) are an important technology for attractive, high efficiency displays and lighting. New applications enabled by OLEDs include flexible^1^, wearable^2^, transparent^3^ and high-resolution displays^4^, as well as efficient and high intensity illumination^5^. The primary impediment to large-scale commercialization of OLEDs, however, is the short operational lifetime of blue-emitting devices^6^. Red and green OLEDs are almost universally based on electrophosphorescent emission due to their 100% internal quantum efficiency (IQE)^7^,^8^ and operational lifetimes of T95>10,000 h which are sufficient for most display and lighting applications^6^. (Here, T*X* is the time elapsed for the luminance to decrease to *X* % of its initial value of *L*_0_=1,000 cd m^−2^ under constant current operation.)

In contrast, the realization of long-lived blue electrophosphorescent OLEDs (PHOLEDs) has not been achieved since its first demonstration in 2001 (ref. 9). Surprisingly, T80 of the blue PHOLEDs with 1931 Commission Internationale de l'Eclairage (CIE) chromaticity coordinates of *y*<0.4 are <10 h^10^,^11^. Even for greenish-blue devices with *y*≥0.4, T80 is <160 h, which is still too small for practical use^12^,^13^,^14^,^15^,^16^. This has led to the use of significantly less efficient fluorescent OLEDs for blue emission. Even so, the lifetime of blue fluorescent OLEDs is insufficient for many applications^17^ and are at least ten times less than state-of-the-art red and green PHOLEDs^18^. In the same vein, the lifetime of green thermally assisted delayed fluorescent OLEDs is only T95=1,300 h^19^, and considerably less for blue.

The short operational lifetime of blue PHOLEDs has been convincingly attributed to annihilation between excited states (that is, exciton–exciton or exciton–polaron) in the device emission layer (EML)^20^,^21^,^22^ that result in a hot (that is, multiply excited) exciton or polaron while the remaining state nonradiatively transitions to the ground state. This process is analogous to Auger recombination in inorganic semiconductor light-emitting diodes and lasers that also has been found to adversely affect device performance^23^. The hot state in the PHOLED EML can attain up to double the energy of the initial excited state (≥6.0 eV). Thus, there is a possibility that their dissipation on dopant or host molecules can induce chemical bond dissociation^24^. Indeed, there are no bonds in organic molecules used in OLEDs that can tolerate the concentration of such a high energy without inducing molecular dissociation. The probability of this reaction increases with twice the excited state energy, and hence is particularly dominant for blue PHOLEDs compared with red and green-emitting analogues.

The key to realizing long-lived blue PHOLEDs is, therefore, to prevent the hot state energies from ever leading to molecular dissociation reactions in the first place. This can be accomplished by reducing bimolecular annihilations, or by bypassing the dissociative processes altogether. In this work, we demonstrate a strategy to thermalize the hot states without damaging the dopant or host molecules. For that purpose, we add an ancillary, protective dopant called an excited state manager into the EML whose triplet exciton energy is higher than the emitting triplets on the dopant. Thus, the manager does not trap excited states, but rather it efficiently returns them to the dopant where they can emit light. Further, by providing exothermic energy transfer pathways from the hot states to the manager, the probability of direct dissociation of the active materials comprising the EML are reduced, or possibly eliminated.

By locating the manager dopant in the region where the triplet excitons have the highest density and thus bimolecular annihilation is most probable, the longest-lived managed blue PHOLEDs achieve T80=334±5 h at *L*_0_=1,000 cd m^−2^ with CIE coordinates of (0.16, 0.30), which is a 3.6±0.1 and 1.9±0.1 times improved lifetime compared to conventional and graded-EML devices^25^ of T80=93±9 and 173±3 h, respectively. Indeed, the lifetime of managed blue PHOLEDs is at least 30 times longer than previously reported blue PHOLEDs with similar colour coordinates^10^,^11^. Our strategy contrasts with previous methods that have employed third components^19^,^26^,^27^, but none of which directly address the siphoning of energy from the most vulnerable constituents of blue PHOLEDs; that is, the dopant and host molecules in the EML. Based on our results, we provide the selection criteria for ideal manager molecules that can enable further improvement in the stability of blue PHOLEDs.

## Results

### Hot excited state management to extend PHOLED lifetime

**Figure 1:**
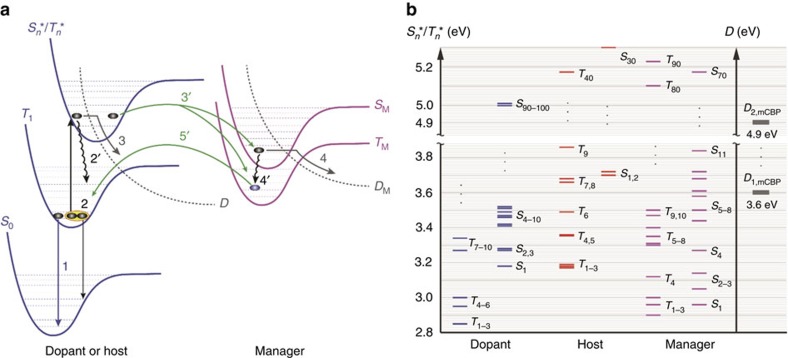
Energetics of the excited states in the PHOLED EML. (**a**) Jablonski diagram of the EML containing the manager. Here, *S*_0_ is the ground state, *T*_1_ is the lowest energy triplet state and S*/T* is a hot singlet/triplet manifold of the dopant or host. *D* represents the dissociative state via the predissociative potential of the EML materials. *S*_M_/*T*_M_ is the lowest singlet/triplet state of the manager. Possible energy-transfer pathways are numbered as follows: (1) radiative recombination, (2) TTA resulting in excitation to S*/T*, (2)′ internal conversion and vibrational relaxation, (3) and (4) dissociative reactions leading to molecular dissociation, (3)′ exothermic Förster energy transfer for singlet-to-singlet transitions, and (3)′ and (5)′ Dexter energy transfer for triplet-to-triplet transitions, and (4)′ intersystem crossing and vibrational relaxation. (**b**) Calculated energies of exciton states for the molecules in the EML (dopant, host and manager) and a few of dissociative states for mCBP used as a host.

Figure 1a shows the Jablonski diagram of an EML containing an excited state manager and the possible relaxation pathways for excitons. The manager can enable the transfer of the hot singlet/triplet state (*S*_n_*/*T*_n_*, where *n*>>1) resulting from triplet–triplet annihilation (TTA, process 2) to the lowest excited state of the manager (*S*_M_/*T*_M_) via process 3′. The hot state can be either an exciton or polaron state resulting from either TTA or triplet–polaron annihilation, respectively^20^.

Figure 1b shows the calculated energy levels of *S*_n_*/*T*_n_* for EML molecules used in this work (see below). When TTA occurs between one or more molecular species in the EML^28^, either the singlet or triplet state is promoted to *S*_n_*/*T*_n_*>5.4 eV. While most hot states rapidly relax to the lowest excited states (*S*_1_/*T*_1_, process 2′), those that have sufficient energy can lead to the chemical bond dissociation via S*_n_/T*_n_→*D* (process 3), where *D* represents dissociative states. Dissociation requires energy in excess of the bond dissociation energy of the excited molecule. For example, bond dissociation energies of weak bonds in the host (*D*_1,mCBP_ and *D*_2,mCBP_, Fig. 1b and Supplementary Note 1) are at 3.5–5 eV above the ground state. TTA can readily supply this energy, while that of the lowest triplet (*T*_1_) is insufficient to induce the dissociation reactions.

By introducing a manager whose energy *S*_M_/*T*_M_ is greater than that of the dopant, excitons formed on, or transferred to the manager can be returned to the dopant for emission. Also, exothermic transfer from *S*_n_*/*T*_n_* to *S*_M_/*T*_M_ is allowed, and damage to these molecules via dissociative reactions (process 3) is reduced provided that the rate for *S*_n_*/*T*_n_*→*S*_M_/*T*_M_ is comparable or higher than *S*_n_*/*T*_n_*→*D*. Since TTA can yield both hot singlets and triplets^29^, the hot state resonantly transfers via a Förster or Dexter process to the manager via process 3. A transferred singlet undergoes vibrational relaxation and Förster transfer back to the lowest dopant singlet state, provided that the manager molecule has a high photoluminescence quantum yield^30^. Alternatively, the thermalized singlet state intersystem crosses to the triplet state (*S*_M_→*T*_M_ via process 4′), which subsequently transfers back to the dopant or host (*T*_M_→*T*_1_) via process 5′. This leads to radiative recombination (process 1), or is recycled back to *S*_n_*/*T*_n_* by a repeat process. It is also possible that the high energy *S*_M_/*T*_M_ state can result in dissociation of the manager itself via *S*_M_/*T*_M_→*D*_M_ (process 4), that is, where the manager serves as a sacrificial additive to the EML. Process 4 is not optimal since the number of effective managers decreases over time, providing less protection for the host and dopant as the device ages. Even in this case, however, the manager can still increase device stability.

From the foregoing discussion, three primary criteria must be met for effective molecular design of the manager: First, the exciton energy of the manager should be higher than lowest exciton states (*S*_1_/*T*_1_) of the dopant; second, the rate of transfer to the manager (process 3′) must be comparable to or higher than that for dissociation (process 3); and third, the manager should be sufficiently stable such that it does not degrade on a time scale short compared to that of the unmanaged device (process 4).

**Figure 2:**
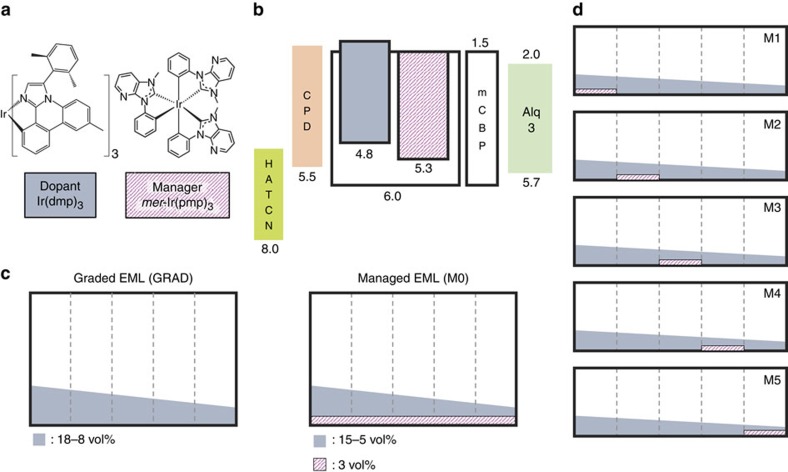
Energy and doping schemes of the PHOLEDs. (**a**) Molecular formulae of Ir(dmp)_3_ and *mer*-Ir(pmp)_3_, used for the dopant and the manager, respectively. (**b**) Energy level diagram of the PHOLED with the manager, denoted ‘managed PHOLED'. Numbers in the figure are energies referred to the vacuum level. (**c**) Doping scheme of the 50 nm-thick EML for the graded-EML and managed PHOLEDs, denoted as GRAD and M0, respectively. GRAD has the dopant graded from 18 to 8 vol% in the mCBP host, while M0 is a similarly graded device but with the 3 vol% of the manager replacing the dopant of the same amount, compared to GRAD, to keep the total doping concentration the same for both devices. (**d**) Managed PHOLEDs M1–M5 have selectively doped 10 nm-thick zones of the EML. The zones have a manager doping of 3 vol% substituting the dopant of the same amount. The other details of the EML are identical to that of GRAD.

**Figure 3:**
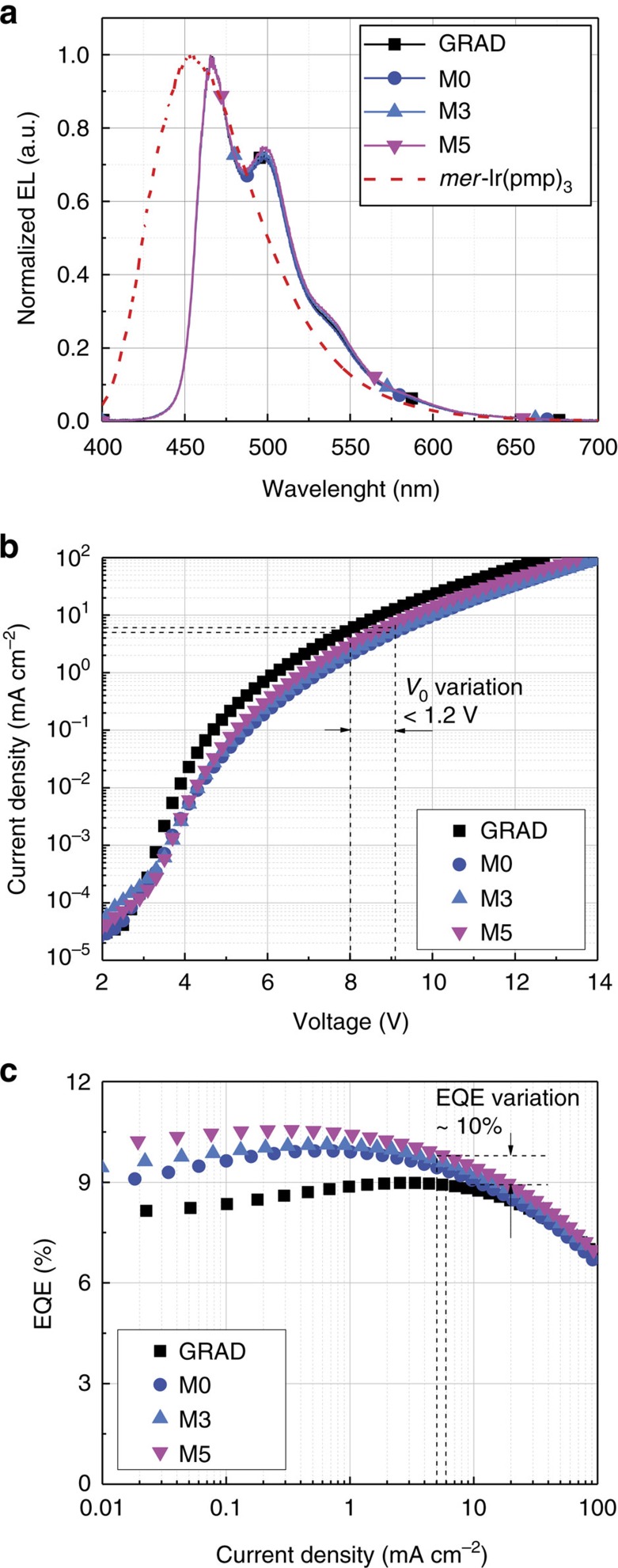
Performance of the PHOLEDs. (**a**), Normalized electroluminescent (EL) spectra of the GRAD and managed PHOLEDs, M0, M3 and M5, measured at a current density of *J*_0_=5 mA cm^−2^. For comparison, the PL spectrum of the manager [*mer*-Ir(pmp)_3_] is also shown. (**b**) Current density–voltage. (**c**) External quantum efficiency (EQE)–current density characteristics of GRAD and selected managed PHOLEDs. Note that between GRAD and the managed PHOLEDs, the absolute difference of the operating voltages (*V*_0_) and EQE at an initial luminance of *L*_0_=1,000 cd m^−2^ for the lifetime test are <1.2 V and 1.0%, respectively.

We introduce meridional-tris-(*N*-phenyl, *N*-methyl-pyridoimidazol-2-yl)iridium (III) [*mer*-Ir(pmp)_3_] as the manager in the PHOLED EML. The EML also consists of the blue dopant, iridium (III) tris[3-(2,6-dimethylphenyl)-7-methylimidazo[1,2-f] phenanthridine] [Ir(dmp)_3_] and the host, 4,4′-bis[*N*-(1-naphthyl)-*N*-phenyl-amino]-biphenyl (mCBP). Figure 2a shows molecular formulae of Ir(dmp)_3_ and *mer*-Ir(pmp)_3_. The manager is characterized by a relatively strong metal–ligand bond^30^ and a glass transition temperature of 136 °C. The triplet energy of *mer*-Ir(pmp)_3_ is 2.8 eV calculated from its peak phosphorescence spectrum (*λ*=454 nm), while its onset is at *E*_T1_≈3.1 eV, higher than that of the dopant of *E*_T1_≈2.8 eV (Fig. 3a). Thus, *mer*-Ir(pmp)_3_ fulfills criterion (i), although both criteria (ii) and (iii) are possibly not met by this molecule. Hence, these complexes have not been optimized for rapid transfer via process 3′. This is a function of the intimate orbital overlap between manager and dopant or host; a property controlled by the steric and orbital characteristics of all molecules involved. Nor is *mer*-Ir(pmp)_3_ particularly stable, which can lead to manager depletion with time (process 4). In spite of these shortcomings, we find significant lifetime improvements for blue PHOLEDs using this manager molecule.

### Performance of managed PHOLEDs

Figure 2b shows the energy level diagram of the managed devices. The lower energy (>1 eV) of the HOMO of the dopant compared with that of the host suggests that hole transfer is predominantly supported by the dopant molecules and only slightly by the manager, while electrons are transported by both the host and the manager having nearly identical lowest unoccupied molecular orbital (LUMO) energies (Supplementary Fig. 2). The EML doping schemes of the control and managed PHOLEDs are given in Fig. 2c (denoted as GRAD and M0, respectively; see Methods). For GRAD, the concentration of the dopant is linearly graded from 18 to 8 vol% from the hole transport layer (HTL) to the electron transport layer interfaces to enable a uniform distribution of excitons and polarons throughout the EML. This structure was previously shown^25^ to reduce bimolecular annihilation, and thereby achieve an extended lifetime compared to conventional, non-graded-EML devices (denoted CONV; see Methods). In device M0, 3 vol% of the manager is uniformly doped across the EML, and the concentration of the dopant is graded from 15 to 5 vol%. To investigate the lifetime dependence on the manager position, the manager is doped at 3 vol% into 10 nm-thick zones at various locations within the 50 nm-thick EML of devices M1–M5, shown in Fig. 2d. Except for the zone with the manager, the remainder of the EMLs for M1–M5 are identical to that of GRAD, keeping the total doping concentrations of all devices the same.

Figure 3a shows the electroluminescence (EL) spectra of GRAD, M0, M3 and M5 measured at a current density of *J*=5 mA cm^−2^. The GRAD and managed PHOLEDs exhibit nearly identical EL spectra with CIE chromaticity coordinates of (0.16, 0.30). This confirms that radiative recombination in managed devices occurs solely on the dopant, while triplets formed on the manager efficiently transfer back to the dopant via process 5′ in Fig. 1.

Figure 3b,c shows the current density–voltage (*J*–*V*) and external quantum efficiency (EQE)–*J* characteristics of GRAD, M0, M3 and M5. Table 1 summarizes properties of their EL characteristics at *L*_0_=1,000 cd m^−2^. The initial operating voltages (*V*_0_) of the managed PHOLEDs (M0–M5) are higher than GRAD by ∼1 V and the voltage at *J*=5 mA cm^−2^ shows a similar trend. This is due to a reduced fraction of the dopant in managed PHOLED EMLs compared to that of GRAD, and due to the manager acting as a hole trap with its HOMO energy of 5.3±0.1 versus 4.8±0.1 eV for the dopant. For example, when a small concentration (<5 vol%) of the manager is added as a substitute of the same amount for the dopant, the device resistance marginally increases (Supplementary Note 2). The EQE for all devices is 9–10%, consistent with the PLQY of the dopant of 44±1% when doped in mCBP at 13 vol%. The EQE of the managed PHOLEDs at *L*_0_=1,000 cd m^−2^ is slightly (<1.0%) higher than that of GRAD, leading to the maximum difference in drive current density of *J*_0_<0.6 mA cm^−2^ needed to achieve the same *L*_0_.

**Figure 4:**
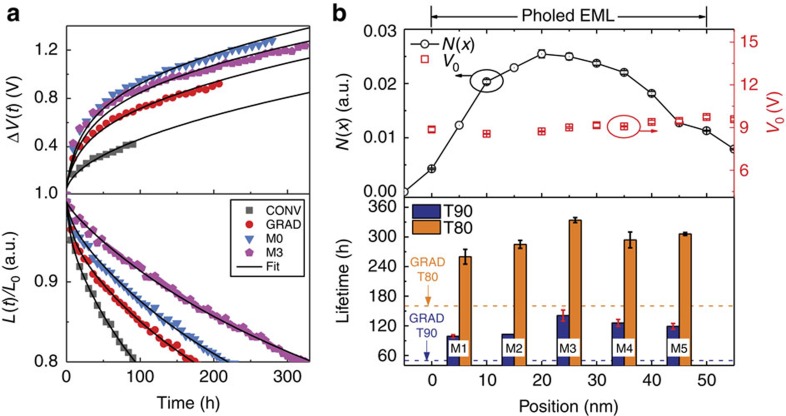
Lifetime and modelling of the PHOLEDs. (**a**) Lifetime characteristics of CONV, GRAD, managed PHOLEDs M0 and M3. Top and bottom show the time evolution of the operating voltage change, Δ*V*(*t*)=*V*(*t*)–*V*_0_, and the normalized luminance degradation, *L*(*t*)/*L*_0_, respectively. Solid lines are fits based on the model in Methods (see fitting parameters in Table 2). (**b**) (Top) Exciton density profile, *N*(*x*), of the PHOLED emission layer (EML) as a function of position, *x*, and operating voltages of the devices using delta-doped sensing layer at *J*=5 mA cm^−2^ (Supplementary Note 3). The origin of the *x*-axis is at the HTL/EML interface. The operating current density results in a luminance of *L*_0_=1,000 cd m^−2^. (Bottom) Lifetimes (T90 and T80) of managed devices (M1–M5) as functions of the position of the managed EML zones. T90 and T80 of the managed devices are compared with those of the GRAD (dotted lines). Note that the variation in lifetime qualitatively follows the exciton density profile, suggesting that placing the manager at the point of highest exciton density results in the longest device lifetime. Error bars represent 1 s.d. for at least three devices.

Figure 4a shows the time evolution of the increase in operating voltage, Δ*V*(*t*)=*V*(*t*)–*V*_0_, and normalized luminance loss, *L*(*t*)/*L*_0_ (*L*_0_=1,000 cd m^−2^) of CONV, GRAD, M0 and M3 under constant current. Table 1 includes the lifetime characteristics (T90, T80 and Δ*V*(*t*)) for all the managed PHOLEDs. Managed PHOLEDs M0–M5 have increased T90 and T80 relative to those of GRAD. For example, the longest-lived device M3 attains T90=141±11 h and T80=334±5 h, corresponding to a 3.0±0.1 and 1.9±0.1 times improvement from those of GRAD and a 5.2±0.2 and 3.6±0.1 times improvement compared with CONV, respectively. Here, T90 and T80 are used to determine the short- and long-term effectiveness of the excited state management.

The upper panel of Fig. 4b shows the measured triplet density profile, *N*(*x*), in the GRAD EML at *J*=5 mA cm^−2^, where *x* is the distance from the EML/HTL interface (see Methods, Supplementary Note 3). The T90 and T80 of M1–M5 versus manager position in the EML are given in the lower panel of Fig. 4b. Note that the variation in lifetime qualitatively follows the exciton density profile. For example, M3 includes the manager at 20 nm<*x*<30 nm, which is at the point of highest exciton density relative to those of other managed devices. Hence, the effectiveness of the manager at this position should be largest, as is indeed observed. Finally, the change in operating voltage, Δ*V*(*t*), required to maintain a constant current is larger for M0–M5 than that of GRAD, while their rate of luminance degradation is reduced. This suggests the formation of polaron traps that have no effect on the luminance.

## Discussion

The degraded molecular products (or defects) can be formed in any and all layers of aged PHOLEDs, but those located in the EML play a dominant role in affecting the device luminance. On the other hand, changes in the operating voltage can arise from defects generated both within and outside the EML. To model the time evolution of the device performance, we consider that two types of charge traps, A and B, with volume densities of *Q*_A_ and *Q*_B_, respectively, are generated by the hot states within the EML. Using thermally stimulated current measurements, we observe an increase in the rate of generation of charge traps and a decreased density of the original transport sites compared to unmanaged devices (Supplementary Note 4). When hot states are generated in blue-emitting devices, all molecular bonds are potentially vulnerable to dissociation by high energy (*E*_S*/T*_∼5.4–6 eV) focused momentarily on a single bond. Dissociated molecular fragments either become neutral species by disproportionation, or they participate in radical addition reactions with neighbouring molecules to form high-molecular-mass products^31^.

To detect degraded molecular products in the aged device, we use laser desorption (LDI)/ionization mass spectroscopy (MS) on fresh and photo-degraded materials. *Mer*-Ir(*pmp*)_3_ shows lower mass defects compared to the parent Ir complex, which are found even in the fresh sample. In degraded *mer*-Ir(*pmp*)_3_, additional higher mass defects are also observed. Similar high and low mass species have also been reported for degraded mCBP. High mass defects have a smaller energy gap than the parent molecule, while the small mass defects show the opposite trend^32^. Details of these investigations will be reported elsewhere.

**Figure 5:**
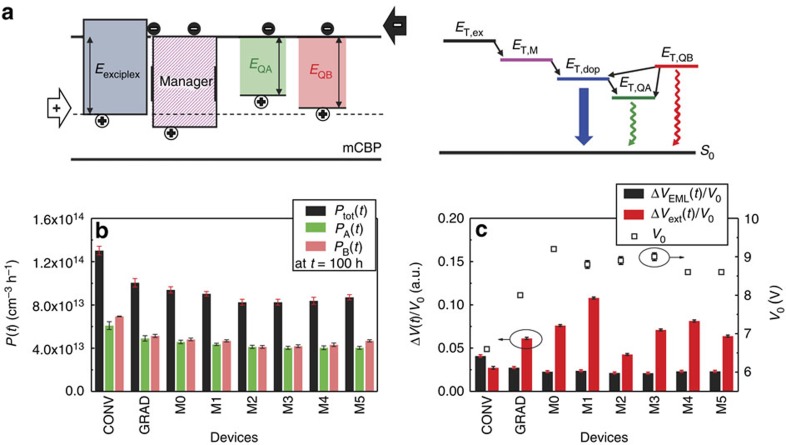
Analysis of the effectiveness of the manager. (**a**) (Left) Energy level diagram of the doped EML along with proposed positions of *Q*_A_ and *Q*_B_. Here, *Q*_A_ and *Q*_B_ are assumed to be hole traps, with *Q*_A_ deeper in the energy gap than *Q*_B_. Holes are transported by the dopant and the manager, and are potentially trapped by *Q*_A_ and *Q*_B_. Electrons are transported by the host and the manager. (Right) Energy diagram of the triplet exciton states in the EML. The sources of triplet excitons in the as-grown device due to charge recombination are twofold: triplet exciplexes (*E*_T,ex_) generated between the host and the dopant, and triplet excitons directly formed on the manager (*E*_T,M_). Both can exothermically transfer to the dopant (*E*_T,dop_). *Q*_A_, the deep hole trap, has a low-energy triplet state that results in exciton quenching (*E*_T,QA_), while *Q*_B_, the shallow trap (*E*_T,QB_), transfers excitons to the lower energy sites. (**b**) Average *Q*_A_ and *Q*_B_ generation rates in the EML, *P*_A_(*t*) and P_B_(*t*), from hot states in CONV, GRAD, and managed PHOLEDs. The total defect generation rate is *P*_tot_(*t*) where *t*=100 h. (**c**) Relative contributions to the voltage rise with respect to *V*_0_ induced by defects within and outside the EML (that is, *Q*_A_+*Q*_B_ and *Q*_ext_, respectively) at *t*=100 h. The separate contributions to the voltage rise, Δ*V*_EML_(*t*)/*V*_0_ and Δ*V*_ext_(*t*)/*V*_0_, along with *V*_0_ are shown. Error bars represent one s.d. for at least three devices.

The small- and large-energy gap defects (relative to the dopant) are identified as the hole traps, *Q*_A_ and *Q*_B_, in Fig. 5a. Both traps are charged when filled, leading to an increase in voltage, Δ*V*(*t*). Shockley–Read–Hall (SRH) nonradiative recombination occurs for holes trapped on *Q*_A_. Likewise, exciton quenching via triplet states at *Q*_A_ results in a decrease in luminance (Fig. 5a). On the other hand, large-energy-gap *Q*_B_ defects can capture excited states that are subsequently transferred to the dopant, and thus do not affect the PHOLED luminance. Note that triplets on the dopant (at energy *E*_T,dop_) are transferred from exciplex states originally formed between the hole on the dopant and the electron on the host (*E*_T,ex_)^25^, as well as from excitons directly formed on the manager (*E*_T,M_).

Based on these considerations, we developed a lifetime model^20^ for fitting both *L*(*t*)/*L*_0_ and Δ*V*(*t*) of CONV, GRAD, and managed PHOLEDs (‘Methods' section). The best fit is provided by assuming that defects generated in the EML are the result of TTA in the devices studied here (Supplementary Note 5). A comparison of lifetime among devices tested at *L*_0_=1,000 cd m^−2^ results in nearly identical initial and steady-state exciton populations, provided that their natural triplet lifetimes and bimolecular annihilation rates are also similar. When GRAD and M3 are driven at *J*_0_=5.3±0.1 mA cm^−2^, the initial luminance levels are 1,000 cd m^−2^ versus 930 cd m^−2^ for M3 and GRAD, respectively. These conditions lead to a slight overestimation of <40 h for T80=173±3 h for GRAD at *L*_0_=1,000 cd m^−2^.

The model also includes polaron traps generated outside of the EML with a density of *Q*_ext_, resulting in the increase of the operating voltage without affecting luminance (‘Methods' section). These traps originate from the degradation of charge transport and blocking layers, all of which are commonly observed in aged devices^24^,^33^,^34^.

Table 2 summarizes the parameters used for fitting the lifetime data for CONV, GRAD and the managed PHOLEDs. The defect generation rates *k*_QA_ and *k*_QB_ are similar for most devices, yielding nearly similar *Q*_A_ and *Q*_B_ in managed PHOLEDs, which are smaller than those in the GRAD and CONV over the same operational period, *t*. For example, *Q*_A_ and *Q*_B_ in M3 at *t*=100 h are (4.9±0.1) and (5.0±0.1) × 10^15^ cm^–3^, while those in GRAD are (5.5±0.2) and (5.7±0.1) × 10^15^ cm^–3^, and those in CONV are (6.6±0.2) and (7.5±0.1) × 10^15^ cm^–3^, respectively. Compared to CONV and GRAD, the reduction in SRH recombination (*k*_Qn_*Q*_A_*n*) and direct exciton quenching (*k*_QN_*Q*_A_*N*) leading to a reduced rate of luminance loss in managed PHOLEDs that is attributed to their lower *Q*_A_. Here, *k*_Qn_ and *k*_QN_ are the reduced Langevin and defect-exciton recombination rates, respectively, and *n* and *N* are the steady-state densities of electrons and excitons, respectively.

The rate of defect formation within the EML is given by 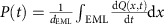. Figure 5b shows the rates for generating *Q*_A_ and *Q*_B_, and *Q*_A_+*Q*_B_ (*P*_A_(*t*), *P*_B_(*t*), and *P*_tot_(*t*), respectively) at *t*=100 h. For example, for CONV, *P*_tot_=(1.3±0.1) × 10^14^ cm^–3^ h^–1^ is reduced to *P*_tot_=(1.0±0.1) × 10^14^ cm^–3 ^h^–1^ for GRAD, and decreases further to *P*_tot_=(0.8±0.1) × 10^14^ cm^–3^ h^–1^ for M3. It is remarkable that only a 15% decrease in the defect formation rate for managed versus graded doping devices leads to a nearly twofold improvement in T80. This result suggests that even a small change in the probability of dissipation of excess energy and the resulting defect density can have large effects on device lifetime, consistent with previous work^20^,^31^,^35^,^36^.

Note that since the luminance loss is primarily due to *Q*_A_, the high *P*_A_ of CONV and GRAD of (6.1±0.4) and (4.9±0.3) × 10^13^ cm^–3^ h^–1^ leads to a luminance of <800 and 850±10 cd m^−2^, respectively, as opposed to that of M3=920±10 cd m^−2^ with *P*_A_=(4.0±0.1) × 10^13^ cm^–3^ h^–1^ at *t*=100 h. On the other hand, M3, M4 and M5 have similar *P*_A_, yielding a luminance of 915±5 cd m^−2^, while *P*_B_ are (4.2±0.1), (4.3±0.2) and (4.7±0.1) × 10^13^ cm^–3^ h^–1^, respectively. This larger variation in *P*_B_ is because *Q*_B_ can return excitons to the dopants where they have a renewed opportunity to luminesce, and thus its effect is small compared to *P*_A_.

The percentage contributions of *k*_Qn_*Q*_A_*n* to the luminance degradation (that is, *k*_Qn_*Q*_A_*n+k*_QN_*Q*_A_*N*) is 90±2% for most devices. This indicates that SRH recombination is the dominant mechanism due to the large density of injected polarons that are lost prior to exciton formation.

The diverse defects with different, distributed energetic characteristics can lead to somewhat larger uncertainties in the hole trapping rate (*k*_Qp_) compared with other parameters extracted from the model (Table 2). Nevertheless, we note that *k*_Qp_ is generally higher for the managed PHOLEDs than that for CONV or GRAD, resulting from energy levels arising from multiple species (Supplementary Note 4). This is offset by the relatively small density of *Q*_A_ in the managed PHOLEDs, additional exciton generation via *Q*_B_, and reduced exciton loss due to the smaller *k*_QN_.

Compared to CONV and GRAD, the managed PHOLEDs have a lower rate of exciton-defect interactions (*k*_QN_), indicating that fewer excitons are eliminated due to the quenching by *Q*_A_ (Fig. 5b). Now, *k*_QN_≅2.0 × 10^–11^ cm^3^ s^−1^ of the aged PHOLEDs is larger by nearly two orders of magnitude than the TTA rate of *k*_TT_≅1.0 × 10^–13^ cm^3^ s^−1^ obtained from the transient PL of the as-grown PHOLED EML. Thus, the reduction of luminance is severely impacted by defect-related exciton loss compared to increased TTA, while the latter process still plays a critical role in triggering molecular dissociation reactions.

Figure 5c shows Δ*V*_EML_(*t*)/*V*_0_ and Δ*V*_ext_(*t*)/*V*_0_ for CONV, GRAD and managed PHOLEDs. These are the relative contributions to the total voltage rise induced by defects within and outside of the EML (that is, *Q*_A_+*Q*_B_ and *Q*_ext_, respectively) at *t*=100 h with respect to *V*_0_. CONV and GRAD have relatively high Δ*V*_EML_(*t*) compared to the managed devices due to the higher defect densities in the EML. The generation rate of *Q*_ext_ that produces Δ*V*_ext_(*t*) is *k*_Qext_, which is generally higher for the managed PHOLEDs than CONV and GRAD. This results from the higher resistivity of the devices due to thick EML, as well as the low hole conductivity in the managed EML. Using an approximation based on space-charge-limited transport^37^, the mobility in the managed EML is reduced by ∼20% compared to that of the GRAD EML. Polaron-induced degradation in the transport layers is accelerated in the managed devices due to the increased polaron density arising from lower hole mobilities^24^,^38^. Thus, while the EML defects (*Q*_A_ and *Q*_B_) are sufficient to accurately model *L*(*t*)/*L*_0_, those formed in other non-luminescent layers of the PHOLEDs (*Q*_ext_) were also included to fully account for Δ*V*(*t*).

The reduced lifetime improvement from 3.0±0.1 to 1.9±0.1 times increases in T90 and T80, respectively, for M3 versus GRAD is due to the degradation of the manager molecules themselves via process 5. Thus, to achieve further increased efficiency, reduced luminance degradation and smaller voltage increase of the devices, manager molecules with improved stability and hole mobility compared with *mer*-Ir(pmp)_3_ are required.

We demonstrate a strategy to dissipate the energy of hot excited states that otherwise lead to dissociative reactions and deteriorate the operational stability of blue PHOLEDs. By introducing excited state manager molecules into the PHOLED EML, we achieve to our knowledge the longest lifetime reported thus far for blue-emitting devices (Supplementary Table 1). We also developed a phenomenological model that establishes the roles and characteristics of defects present in the device. Our findings emphasize the importance of excited state management or similar approaches to further improve the lifetime of blue PHOLEDs. While such approaches based on an understanding of the fundamental underlying processes leading to device failure are essential, they must be accompanied by the development of highly stable dopants, managers, hosts and transport materials; a challenge made all the more difficult by the very wide energy gaps required for blue PHOLEDs.

## Methods

### Device fabrication and characterization

PHOLEDs were grown by vacuum sublimation in a chamber with a base pressure of 4 × 10^−7^ Torr on pre-patterned indium-tin-oxide (ITO) glass substrates (VisionTek Systems Ltd., United Kingdom). The device and the structures of GRAD and managed PHOLEDs are as follows: 70 nm ITO anode/5 nm dipyrazino[2,3,-f:2′,3′-h]quinoxaline 2,3,6,7,10,11-hexacarbonitrile (HATCN) hole injection layer/10 nm *N,N*′-Di(phenyl-carbazole)-*N,N*′-bis-phenyl-(1,1′-biphenyl)-4,4′-diamine (CPD) HTL/50 nm EML/5 nm mCBP:Ir(dmp)_3_ 8 vol% exciton blocking layer/5 nm mCBP hole blocking layer/25 nm tris-(8-hydroxyquinoline)aluminium (Alq_3_) electron transport layer/1.5 nm hydroxyquinolato-Li (Liq) electron injection layer/100 nm Al cathode. The conventional PHOLED (CONV) has the following structure^20^,^25^: 5 nm HATCN/30 nm CPD/35 nm 13 vol% Ir(dmp)_3_ uniformly doped in mCBP/5 nm mCBP/25 nm Alq_3_/1.5 nm Liq/100 nm Al. The device area is 2 mm^2^ defined by the intersection of a 1 mm wide ITO strip and an orthogonally positioned 2 mm wide metal cathode patterned by deposition through a shadow mask. HATCN and Alq_3_ were purchased from Luminescence Technology Corporation (Taiwan), CPD was from P&H Technology (South Korea), mCBP and Ir(dmp)_3_ were provided by Universal Display Corporation (Ewing, NJ, USA) and *mer*-Ir(pmp)_3_ was synthesized following previous methods^30^. The *J–V–L* characteristics of the PHOLEDs were measured^39^ using a parameter analyzer (Hewlett-Packard, HP4145) and a calibrated Si-photodiode (Thorlab, FDS1010-CAL). The PHOLED emission spectra were recorded using a calibrated spectrometer (OceanOptics, USB4000). For lifetime tests, PHOLEDs were operated at constant current (Agilent, U2722) and the luminance and voltage data were automatically collected (Agilent, 34972A). Errors quoted for the measured electroluminescent and lifetime characteristics (*J*_0_, *V*_0_, EQE, T90, T80 and Δ*V*(*t*)) are s.d.'s taken from a population of from three devices.

### Exciton profile measurement

The exciton density profile, *N*(*x*), was measured across the EML by inserting ultrathin (∼1 Å) red phosphorescent (iridium (III) bis (2-phenylquinolyl-N, C^2′^) acetylacetonate (PQIr)) sensing layers at different locations within the EML in a series of blue PHOLEDS^40^,^40^. The integrated emission intensities of PQIr and Ir(dmp)_3_ at *J*_0_ are converted into the number of excitons at *x* via:


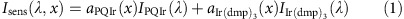


where *I*_sens_(*λ*, *x*) is the emission intensity consisting of the combined spectra of Ir(dmp)_3_ (*I*_Ir(dmp)3_(*λ*)) and PQIr (*I*_PQIr_(*λ*)). The relative weights of *a*_PQIr_(*x*) and *a*_Ir(dmp)3_(*x*), respectively, were used. Then, the outcoupled exciton density, *η*_out_(*x*)*N*(*x*), is equal to the relative number of excitons emitting on the PQIr at *x* as:


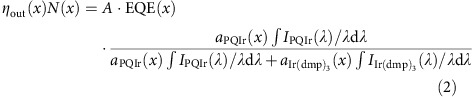


Here, EQE(*x*) is external quantum efficiency of the device with the sensing layer at *x*; thus the right-hand side of equation (2) gives the relative number of excitons at position *x*. Also, 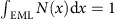, and *η*_out_(*x*) is the outcoupling efficiency calculated as the fraction of outcoupled light emitted at *x* based on Green's function analysis^42^. The Förster transfer length of ∼3 nm^25^ limits the spatial resolution of the measurement.

Since the thickness of delta-doped PQIr is less than a monolayer, PQIr molecules are spatially dispersed to avoid emission loss by concentration quenching. A delta-doped sensing layer only slightly affects the charge transport as opposed to previously used 1–2 nm-thick, doped layers^25^. This leads to a variation in operating voltages at *J*_0_ of <0.5 V among all sensing devices (see also the upper panel of Fig. 4b and Supplementary Note 3).

### Mass spectrometry measurement

Materials used in the PHOLEDs were prepared in N_2_-filled encapsulated vials. They were photodegraded by the laser irradiation at *λ*=442 nm for >5 h. For the LDI–MS measurement, the material was dissolved in toluene/THF, and the solution is placed onto the target plate and subsequently evaporated. The Bruker Autoflex Speed mass spectrometer is run in reflection mode. The spectrometer was calibrated with a series of known peptides and matrix peaks. Mass spectra of degraded materials were compared to those of their pristine counterparts.

### Lifetime degradation model

The rate equations for holes (*p*), electrons (*n*) and excitons (*N*) are:


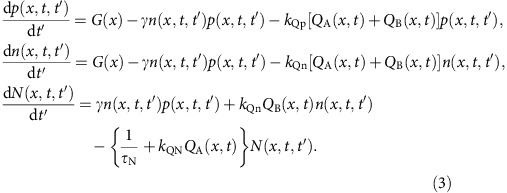


There are two different time scales: *t'* is the duration of charge transport and energy transfer (∼μs) and *t* is the device degradation time (∼h) due to the formation of defects, *Q*_A_(*x*,*t*) and *Q*_B_(*x*,*t*). The triplet decay lifetime is *τ*_N_=1.4±0.1 μs, obtained from the transient PL decay of thin-film EMLs of the GRAD and managed PHOLEDs. Also, 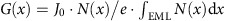 is the generation rate of excitons due to charge injection at current *J*_0_, 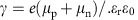 is the Langevin recombination rate, where *e* is the elementary charge, *μ*_n_ and *μ*_p_ are the electron and hole mobilities in the EML, respectively, and *ɛ*_0_ and *ɛ*_r_∼3 are the vacuum and relative permittivities, respectively. It follows that 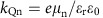 is the reduced Langevin recombination rate describing the recombination of immobile trapped holes and mobile electrons.

The trap densities, *Q*_A_ and *Q*_B_, resulting from the TTA increase at rates *k*_QA_ and *k*_QB_ are given by:


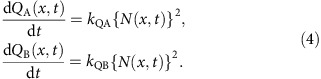


Equation (3) is solved in steady state (*t′*→∞), yielding 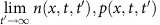 and *N*(*x*,*t*,*t′*)=*n*(*x*,*t*), *p*(*x*,*t*) and *N*(*x*,*t*), respectively. This set of equations is numerically solved with *Q*_A_(*x*,*t*) and *Q*_B_(*x*,*t*) to fit both the luminance loss and voltage rise as a function of *t* using:


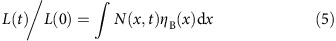


and


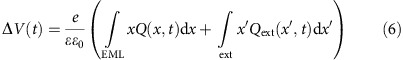


Here, *η*_B_(*x*) is the outcoupling efficiency of the excitons emitted at *x* and *Q*_ext_(*x′*,*t*) is introduced to account for the voltage rise caused by traps present outside the EML. The uniqueness of the fit that yields parameters, *k*_QN_, *k*_Qp_, *k*_QA_, *k*_QB_ and *k*_Qext_, has been tested, with results in Supplementary Note 5.

Note that when extracting *k*_Qext_ and thus Δ*V*_ext_(*t*) from the fits, the polaron densities in the EML at *J*_0_ are used. However, *k*_Qext_ should more accurately reflect the polaron densities in the transport layers due to charge trapping by *Q*_ext_, and thus, a reduction in layer conductivity. This simplifying assumption leads to its large variation among devices compared with other parameters. Initial values of *Q*_A_, *Q*_B_ and *Q*_ext_ are set at 10^15^ (cm^–3^), which accurately traces the time evolution of Δ*V*(*t*) and converge to their final values after the iteration of the least-square algorithm.

### Data availability

The data that support the findings of this study are available from the authors upon request.

## Additional information

**How to cite this article:** Lee, J. *et al*. Hot excited state management for long-lived blue phosphorescent organic light-emitting diodes. *Nat. Commun.* **8**, 15566 doi: 10.1038/ncomms15566 (2017).

**Publisher's note:** Springer Nature remains neutral with regard to jurisdictional claims in published maps and institutional affiliations.

## Supplementary Material

Supplementary Information Supplementary Figures, Supplementary Notes and Supplementary References

## Figures and Tables

**Table 1:** Electroluminescent and lifetime characteristics for CONV, GRAD and managed PHOLEDs (M0–M5) at *L*_0_=1,000 cd m^−2^.

**Device**	***J***_**0**_ **(mA cm**^**−2**^**)**	**EQE (%)**	***V***_**0**_ **(V)**	CIE*	**T90 (h)**	**T80 (h)**	**Δ*****V*****(T90) (V)**	**Δ*****V*****(T80) (V)**
CONV	6.7±0.1	8.0±0.1	6.6±0.0	[0.15, 0.28]	27±4	93±9	0.3±0.1	0.4±0.1
GRAD	5.7±0.1	8.9±0.1	8.0±0.0	[0.16, 0.30]	47±1	173±3	0.6±0.1	0.9±0.1
M0	5.5±0.1	9.4±0.1	9.2±0.0	[0.16, 0.30]	71±1	226±9	0.9±0.1	1.2±0.1
M1	5.4±0.1	9.5±0.1	8.8±0.1	[0.16, 0.29]	99±3	260±15	1.2±0.1	1.6±0.1
M2	5.4±0.1	9.3±0.0	8.9±0.1	[0.16, 0.31]	103±0	285±8	0.7±0.1	1.0±0.1
M3	5.3±0.1	9.6±0.0	9.0±0.1	[0.16, 0.30]	141±11	334±5	1.1±0.1	1.5±0.2
M4	5.2±0.1	9.6±0.2	8.6±0.0	[0.16, 0.31]	126±7	294±16	1.0±0.1	1.3±0.1
M5	5.1±0.1	9.9±0.1	8.6±0.0	[0.16, 0.31]	119±6	306±3	0.9±0.1	1.2±0.1

EQE, external quantum efficiency.

^*^Measured at current density of *J*=5 mA cm^−2^.

Errors for the measured values are s.d. from at least three devices.

**Table 2:** Model parameters for the lifetime model for CONV, GRAD and managed PHOLEDs.

**Device**	***k***_**QN**_ **(10**^−**11**^** cm**^**3**^** s**^**−1**^**)**	***k***_**Qp**_ **(10**^−**7**^** cm**^**3**^** s**^**−1**^**)**	***k***_**QA**_ **(10**^−**21**^** cm**^**3**^** s**^**−1**^**)**	***k***_**QB**_ **(10**^−**21**^** cm**^**3**^** s**^**−1**^**)**	***k***_**Qext**_ **(10**^−**21**^** cm**^**3**^** s**^**−1**^**)**
CONV	3.3±0.4	0.7±0.2	0.9±0.1	1.0±0.1	0.06±0.01
GRAD	2.3±0.2	0.9±0.2	0.9±0.1	1.0±0.1	0.2±0.01
M0	2.3±0.1	1.3±0.2	1.0±0.1	1.0±0.1	0.5±0.1
M1	2.1±0.1	1.6±0.2	0.9±0.1	1.0±0.1	0.8±0.1
M2	1.9±0.1	3.0±0.7	0.9±0.1	0.9±0.1	0.5±0.1
M3	1.9±0.1	3.0±0.8	0.9±0.1	0.9±0.1	1.0±0.3
M4	2.1±0.1	2.1±0.5	0.9±0.1	1.0±0.1	0.8±0.2
M5	2.0±0.1	0.9±0.1	0.9±0.1	1.0±0.1	0.3±0.1

Errors for the model parameters are the 95% confidence interval for fit.

## References

[b1] Kim S. . Low-power flexible organic light-emitting diode display device. Adv. Mater. 23, 3511–3516 (2011). 21735486 10.1002/adma.201101066

[b2] Kim S.-W. Organic light emitting diode display. US patent 2014/0312319 A1 (2014).

[b3] Lee C. & Park G. Transparent display panel and transparent organic light emitting diode diplay device including the same. US patent 2016/0055794 A1 (2014).

[b4] Jung Y. K. . 52-3: distinguished paper: 3 stacked top emitting white OLED for high resolution OLED TV. SID Symp. Dig. Tech. Pap. 47, 707–710 (2016).

[b5] Lee J., Slootsky M., Lee K., Zhang Y. & Forrest S. R. An electrophosphorescent organic light emitting concentrator. Light Sci. Appl. 3, e181 (2014).

[b6] Hack M., Brown J. J., Weaver M. S. & Premutico M. Lifetime OLED display. US patent 9257665 B2 (2016).

[b7] Baldo M. A. . Highly efficient phosphorescent emission from organic electroluminescent devices. Nature 395, 151–154 (1998).

[b8] Baldo M. A., Lamansky S., Burrows P. E., Thompson M. E. & Forrest S. R. Very high-efficiency green organic light-emitting devices based on electrophosphorescence. Appl. Phys. Lett. 75, 4–6 (1999).

[b9] Adachi C. . Endothermic energy transfer: A mechanism for generating very efficient high-energy phosphorescent emission in organic materials. Appl. Phys. Lett. 79, 2082–2084 (2001).

[b10] Zhuang J. . Highly efficient phosphorescent organic light-emitting diodes using a homoleptic iridium(III) complex as a sky-blue dopant. Org. Electron. 14, 2596–2601 (2013).

[b11] Klubek K. P., Dong S.-C., Liao L.-S., Tang C. W. & Rothberg L. J. Investigating blue phosphorescent iridium cyclometalated dopant with phenyl-imidazole ligands. Org. Electron. 15, 3127–3136 (2014).

[b12] Oh C. S., Choi J. M. & Lee J. Y. Chemical bond stabilization and exciton management by CN modified host material for improved efficiency and lifetime in blue phosphorescent organic light-emitting diodes. Adv. Opt. Mater. 4, 1281–1287 (2016).

[b13] Kang Y. J. & Lee J. Y. High triplet energy electron transport type exciton blocking materials for stable blue phosphorescent organic light-emitting diodes. Org. Electron. 32, 109–114 (2016).

[b14] Zhang L. . Highly efficient blue phosphorescent organic light-emitting diodes employing a host material with small bandgap. ACS Appl. Mater. Interfaces 8, 16186–16191 (2016). 27281124 10.1021/acsami.6b01304

[b15] Seo J.-A. . Long lifetime blue phosphorescent organic light-emitting diodes with an exciton blocking layer. J. Mater. Chem. C 3, 4640–4645 (2015).

[b16] Jeon S. K. & Lee J. Y. Four times lifetime improvement of blue phosphorescent organic light-emitting diodes by managing recombination zone. Org. Electron. 27, 202–206 (2015).

[b17] Huang H.-L., Balaganesan B., Fu Y.-H., Lin H.-Y. & Chao T.-C. P-159: electron transporting materials for highly efficient and long lifetime blue OLED devices for display and lighting applications. SID Symp. Dig. Tech. Pap. 47, 1722–1724 (2016).

[b18] D'Andrade B., Esler J., Lin C., Weaver M. & Brown J. 61.5L: late-news paper: extremely long lived white phosphorescent organic light emitting device with minimum organic materials. SID Symp. Dig. Tech. Pap. 39, 940–942 (2008).

[b19] Tsang D. P.-K. & Adachi C. Operational stability enhancement in organic light-emitting diodes with ultrathin Liq interlayers. Sci. Rep. 6, 22463 (2016). 26926237 10.1038/srep22463 PMC4772539

[b20] Giebink N. C. . Intrinsic luminance loss in phosphorescent small-molecule organic light emitting devices due to bimolecular annihilation reactions. J. Appl. Phys. 103, 44509 (2008).

[b21] Giebink N. C., D'Andrade B. W., Weaver M. S., Brown J. J. & Forrest S. R. Direct evidence for degradation of polaron excited states in organic light emitting diodes. J. Appl. Phys. 105, 124514 (2009).

[b22] Wang Q. & Aziz H. Degradation of organic/organic interfaces in organic light-emitting devices due to polaron–exciton interactions. ACS Appl. Mater. Interfaces 5, 8733–8739 (2013). 23937296 10.1021/am402537j

[b23] Sadi T., Kivisaari P., Oksanen J. & Tulkki J. On the correlation of the Auger generated hot electron emission and efficiency droop in III-N light-emitting diodes. Appl. Phys. Lett. 105, 91106 (2014).

[b24] Schmidbauer S., Hohenleutner A. & König B. Chemical degradation in organic light-emitting devices: mechanisms and implications for the design of new materials. Adv. Mater. 25, 2114–2129 (2013). 23450816 10.1002/adma.201205022

[b25] Zhang Y., Lee J. & Forrest S. R. Tenfold increase in the lifetime of blue phosphorescent organic light-emitting diodes. Nat. Commun. 5, 5008 (2014). 25254492 10.1038/ncomms6008

[b26] Hong S., Kim J. W. & Lee S. Lifetime enhanced phosphorescent organic light emitting diode using an electron scavenger layer. Appl. Phys. Lett. 107, 41117 (2015).

[b27] Hsin M.-H. . P-161: 89.3% lifetime elongation of blue TTA-OLED with assistant host. SID Symp. Dig. Tech. Pap. 47, 1727–1729 (2016).

[b28] Baldo M. A., Adachi C. & Forrest S. R. Transient analysis of organic electrophosphorescence. II. Transient analysis of triplet-triplet annihilation. Phys. Rev. B 62, 10967–10977 (2000).

[b29] Bachilo S. M. & Weisman R. B. Determination of triplet quantum yields from triplet−triplet annihilation fluorescence. J. Phys. Chem. A 104, 7711–7714 (2000).

[b30] Lee J. . Deep blue phosphorescent organic light-emitting diodes with very high brightness and efficiency. Nat. Mater. 15, 92–98 (2016). 26480228 10.1038/nmat4446

[b31] Kondakov D. Y., Lenhart W. C. & Nichols W. F. Operational degradation of organic light-emitting diodes: mechanism and identification of chemical products. J. Appl. Phys. 101, 24512 (2007).

[b32] Sandanayaka A. S. D., Matsushima T. & Adachi C. Degradation mechanisms of organic light-emitting diodes based on thermally activated delayed fluorescence molecules. J. Phys. Chem. C 119, 23845–23851 (2015).

[b33] Adamovich V. I., Weaver M. S. & D'Andrade B. W. Long lifetime phosphorescent organic light emitting device (OLED) structures. US patent 8866377 B2 (2014).

[b34] Kwong R. C. . High operational stability of electrophosphorescent devices. Appl. Phys. Lett. 81, 162–164 (2002).

[b35] Winter S., Reineke S., Walzer K. & Leo K. Photoluminescence degradation of blue OLED emitters. *Proc. SPIE* **6999**, 69992N (2008).

[b36] Fujimoto H. . Influence of material impurities in the hole-blocking layer on the lifetime of organic light-emitting diodes. Appl. Phys. Lett. 109, 243302 (2016).

[b37] Lampert M. A. Simplified theory of space-charge-limited currents in an insulator with traps. Phys. Rev. 103, 1648–1656 (1956).

[b38] Xia S. C., Kwong R. C., Adamovich V. I., Weaver M. S. & Brown J. J. in *Proceedings of the 45th IEEE International Annual Reliability Physics Symposium*, 253–257 (2007).

[b39] Forrest S. R., Bradley D. D. C. & Thompson M. E. Measuring the efficiency of organic light-emitting devices. Adv. Mater. 15, 1043–1048 (2003).

[b40] Erickson N. C. & Holmes R. J. Investigating the role of emissive layer architecture on the exciton recombination zone in organic light-emitting devices. Adv. Funct. Mater. 23, 5190–5198 (2013).

[b41] Coburn C., Lee J. & Forrest S. R. Charge balance and exciton confinement in phosphorescent organic light emitting diodes. Adv. Opt. Mater. 4, 889–895 (2016).

[b42] Celebi K., Heidel T. D. & Baldo M. A. Simplified calculation of dipole energy transport in a multilayer stack using dyadic Green's functions. Opt. Express 15, 1762–1772 (2007). 19532414 10.1364/oe.15.001762

