# Supplementary material for: Hot excited state management for long-lived blue phosphorescent organic light-emitting diodes
Source: Nat Commun. 2017 May 31;8:15566. doi: 10.1038/ncomms15566 (PMC5460033; doi:10.1038/ncomms15566)
Supplement: Supplementary Information — Supplementary Figures, Supplementary Notes and Supplementary References [file ncomms15566-s1.pdf]

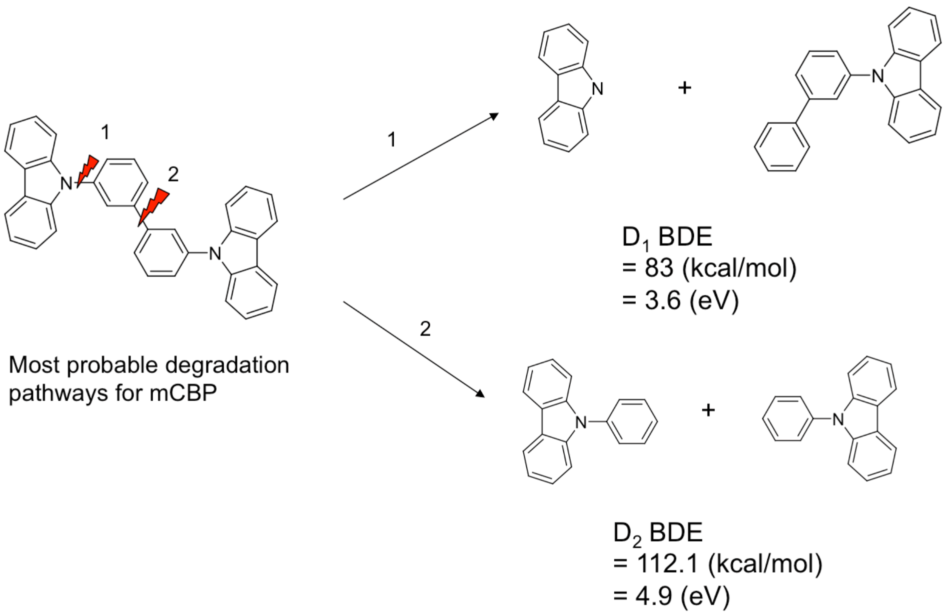

Supplementary Figure 1 | Potential routes of chemical bond dissociation for mCBP.

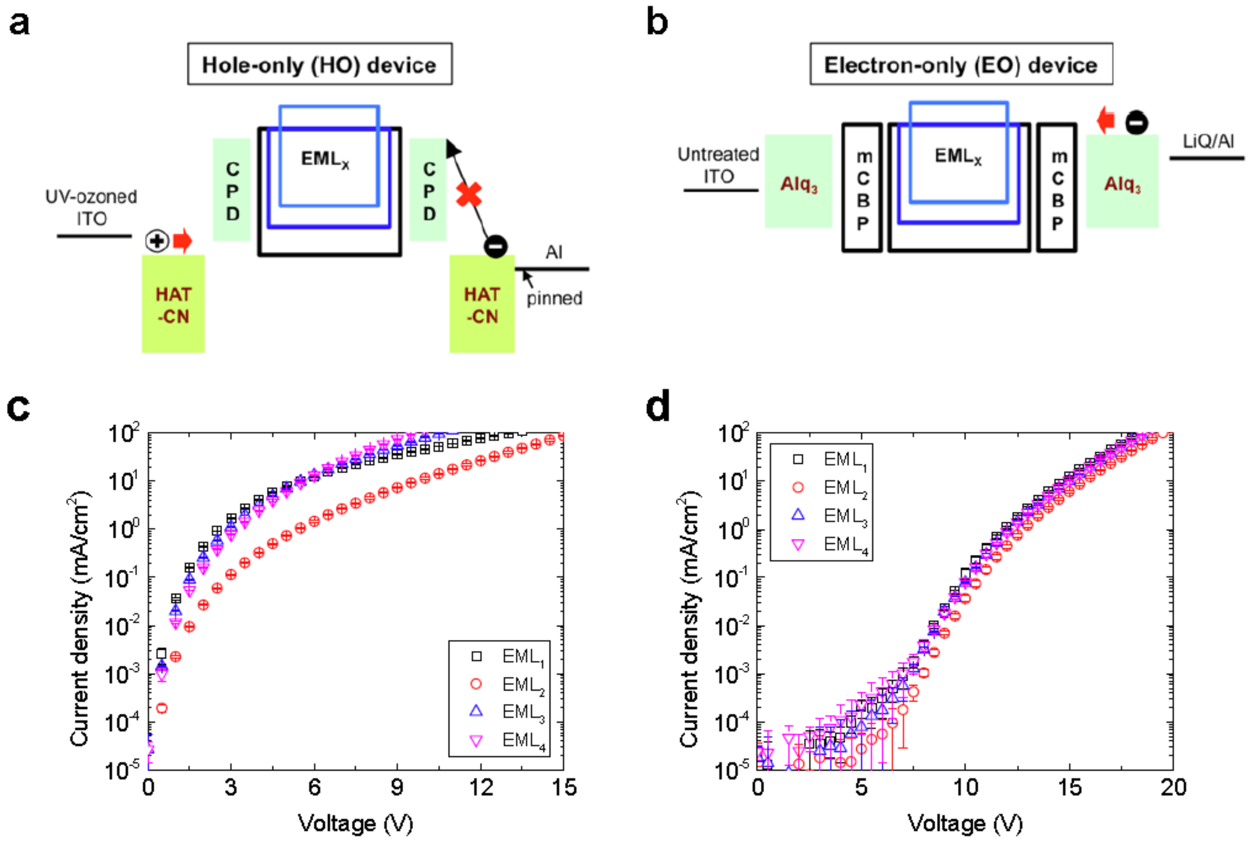

Supplementary Figure 2 | Emission layer charge transport characteristics. **a**,**b**. Schematic structures of hole-only (HO) and electron-only (EO, right) devices. Both devices have the same EML structure comprising the blue dopant [Ir(dmp)$_3$] and manager [*mer*-Ir(pmp)$_3$] doped at varying concentrations into the host (mCBP). **c**,**d**. Current density–voltage characteristics of HO and EO devices with the different EML structures.

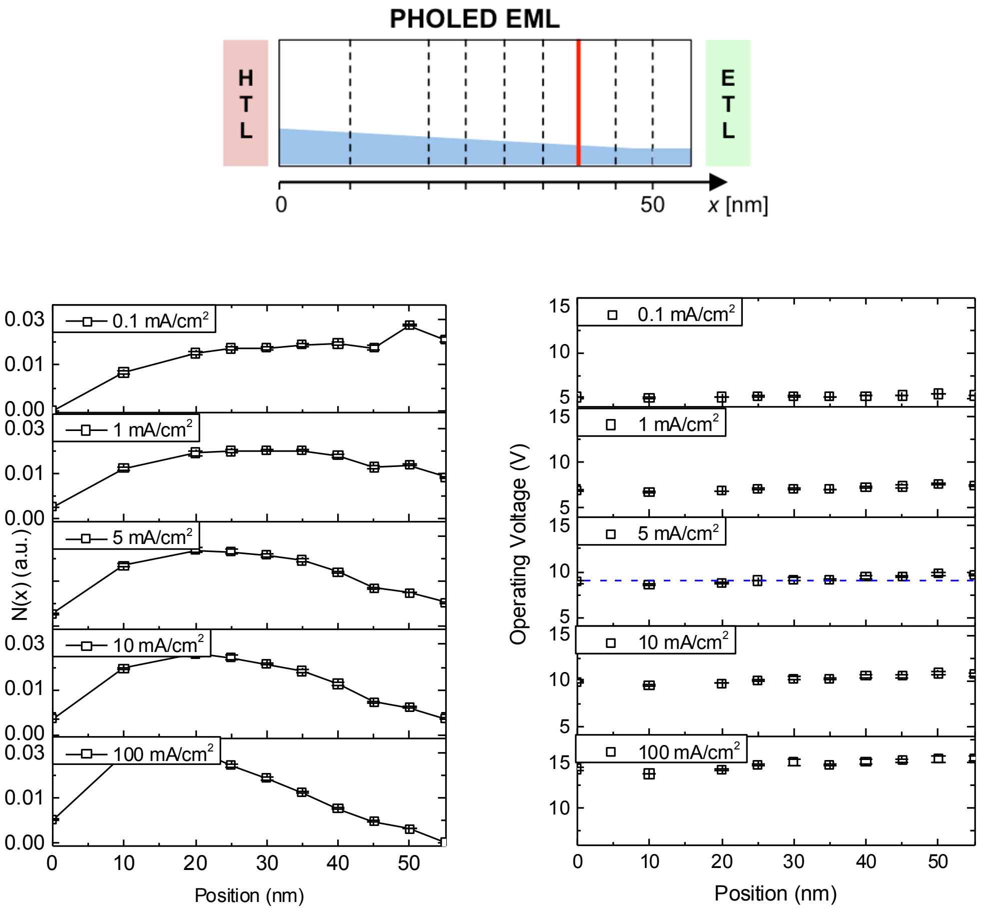

Supplementary Figure 3 | Local exciton density profile in the EML using delta doped sensing layers. (Top) Schematic diagram for probing local triplet density in the EML. A 1Å-thick red sensing layer, iridium (III) bis (2-phenylquinolyl-N, $C^{2'}$) acetylacetonate (PQIr), is inserted at positions, *x*. (left) Local exciton density profile, $N(x)$, of the EML of the GRAD PHOLED, where *x* is the distance from the interface between the hole transport layer and the EML. $N(x)$ is measured at current densities of J = 0.1, 1, 5, 10, and 100 mA/cm^2 (from top to bottom). (right) Operating voltages of the PHOLEDs having the sensing layer at *x* are measured at different *J* (see **Methods** in text for details).

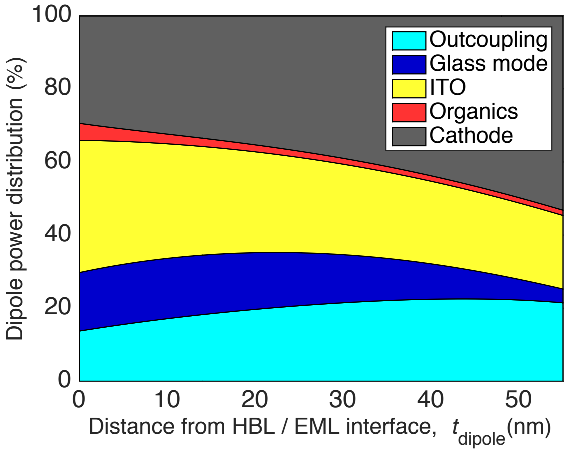

Supplementary Figure 4 | Calculated power distribution of a dipole in the blue PHOLED EML as a function of the distance of the dipole from the mCBP HBL/EML interface, t_{dipole}.

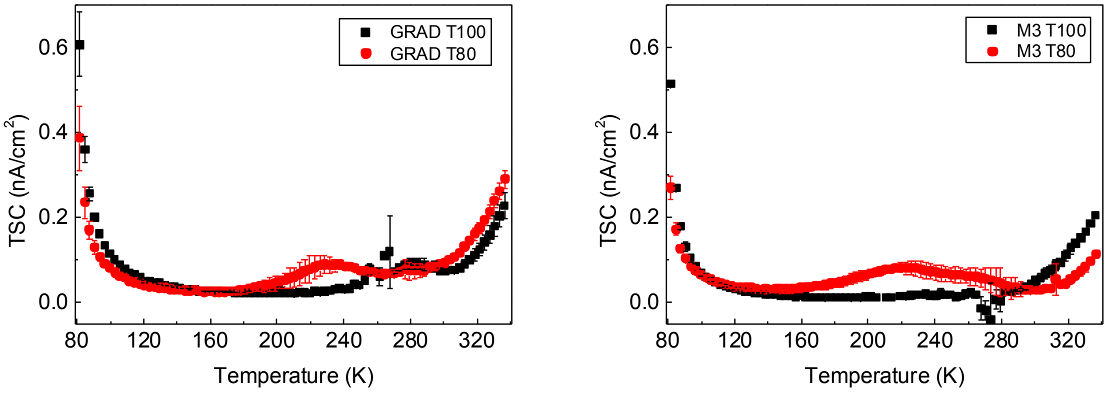

Supplementary Figure 5 | TSC of GRAD and M3 PHOLEDs measured at their T100 and T80.

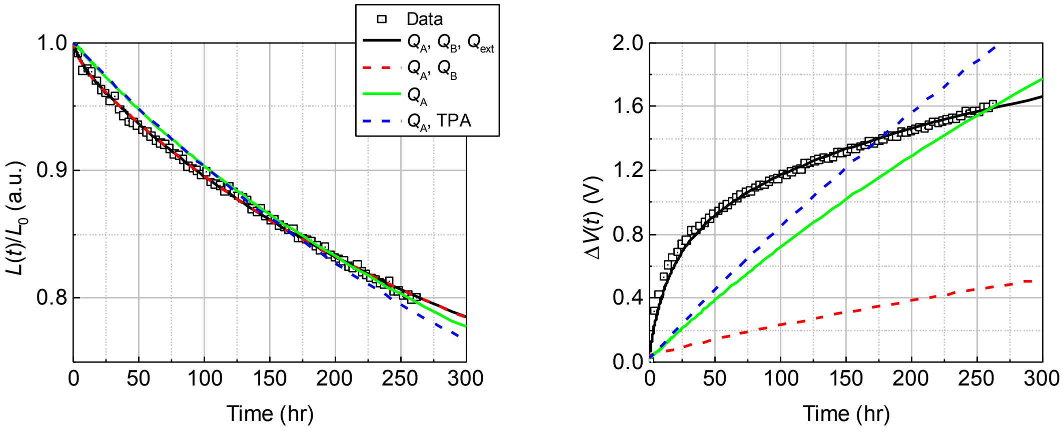

Supplementary Figure 6 | Lifetime model fit based on different combinations of the defects (solid and dashed lines). The squares show the time evolution of the measured luminance (left) and operational voltage (right).

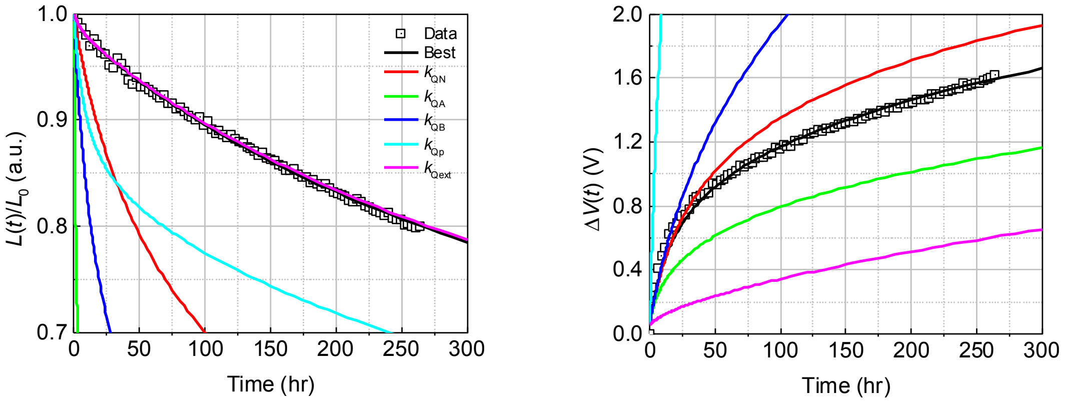

Supplementary Figure 7 | Lifetime model fit by changing a single parameter by an order of magnitude smaller or larger than the one providing the best fit, while fixing others. The squares are data for the time evolution of the luminance (left) and operational voltage (right).

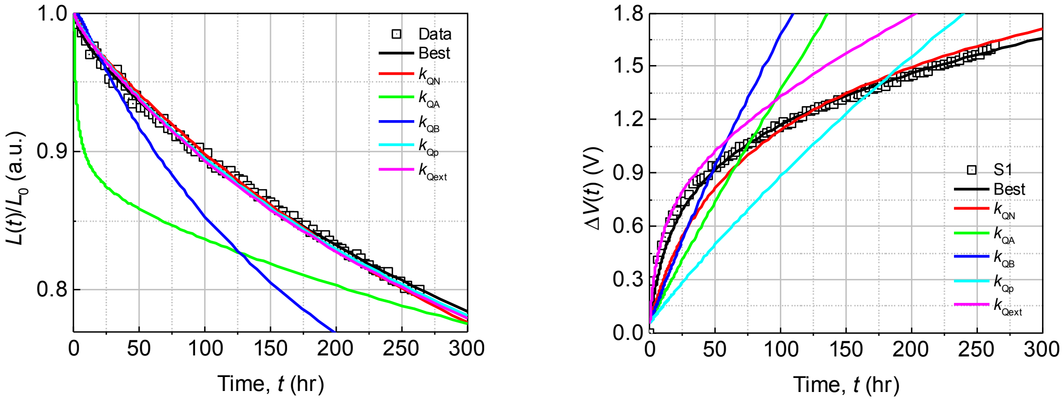

Supplementary Figure 8 | Lifetime model fit by setting a single parameter to smaller or larger values by an order of magnitude from its final value, while varying other parameters. The squares are data for the time evolution of the luminance (left) and operational voltage (right).

Supplementary Table 1 | Lifetime comparisons of reported cyan PHOLEDs

Dopant	†J_0 (mA/cm^2)	EQE (%)	Luminance (cd/m^2)	†V_0 (V)	CIE	λ_{onset} / λ_{peak} (nm)	†L_0 (cd/m^2)	Lifetime (hr)	Ref.
$Ir(dmp)_3$	5.3	9.6±0.0	1,000	9.0±0.1	(0.16, 0.30)	440 / 462	1,000	334±5 (T80)	This work
$Ir(dbi)_3$	N/A	24.3 / 23.0	Max / 1,000	~6.2	(0.19, 0.44)	440 / 475	1,000	15 (T80)	[8]
$Ir(dbi)_3$	~5	7	1,000	~8.5	(0.19, 0.44)	440 / 475	1,000	7 (T80)	[9]
$Ir(iprpmi)_3$	N/A	19.9 / 19.6	Max / 1,000	7.82	(0.17, 0.40)	450 / 474	1,000	~20 (T80)	[10]
$Ir(dbi)_3$	~6	~14 / ~11	Max / 1,000	10	(0.19, 0.44)	440 / 475	500	~60 (T80)	[11]
FIrpic & Pyrene-CN	60.1	~ 2.5	1,000	~8	(0.15, 0.13)	430/ 455	500	~1 (T80)	[12]
FIrpic	~5	13.5 / 12.6	Max / 1,000	7.4	(0.16, 0.36)	435 / 467	1,000	0.1 (T50)	[13]
$Ir(dbi)_3$	~3	23.1 / 20.1	Max / 1,000	5.6	(0.19, 0.44)	440 / 475	1,000	5.8 (T50)	[13]
Ir(ipripmi)	N/A	~ 14	Max	N/A	(0.17, 0.37)	450 / 474	5 mA/cm^2	30 (T70)	[14]
PtNON	~12	10.7 / 9.1	Max / 1,000	~13	(0.17, 0.32)	420 / 476	1,000	*335 (T80)	[15]
$Ir(dbi)_3$	N/A	~ 8 / 4	Max / 1,000	9.25	(0.19, 0.44)	440 / 475	1,000	154 (T80)	[16]
$Ir(dmp)_3$	N/A	N/A	N/A	N/A	(0.15, 0.25)	440 / 462	15 mA/cm^2	225 (T50)	[17]

† J_0, V_0, and L_0 are the operating current density, initial voltage, and luminance, respectively, for the lifetime test.

* Extrapolated using the relation, $T(1{,}000\ cd/m^2) = T(L_0)\times(L_0/1{,}000)^{1.7}$.

Supplementary Note 1 | Calculation of dissociative state energy

Supplementary Figure 1 shows the potential routes of chemical bond dissociation for the mCBP host. Bond dissociation energies of a *C–N* bond in 9-phenylcarbazole moieties and their connecting *C–C* bond are BDE = 3.6 eV and 4.9 eV, respectively, and corresponding repulsive states are denoted as D_1 and D_2. The lowest triplet excitons (T_1 < 3.0 eV) in EML molecules do not have sufficient energy to trigger the dissociative reaction, whereas hot excited states as a result of TTA, S_n^*/ T_n^* where n >>1, can readily surmount required energies of any *D* states and couple to them. Direct evidence has been found for this and several other dissociative TTA-initiated reactions of the EML materials. Details of our methods are in Sec. 5, with a full report of these results currently in preparation.

Supplementary Note 2 | Charge transport characteristics of the emissive layer

It has previously been shown that electrons are transported by the host while holes are transported by the blue dopant[1] in EML_1. This experiment is performed to identify how the charge transport characteristics are modified by including the manger in the EML_1. The structures of hole-only (HO) and electron-only (EO) devices are as follow (**Supplementary Figure 2**):

<u>HO devices</u> – ITO (ultraviolet-ozone treated) / 5 nm HATCN / 10 nm CPD / 40 nm EML_x / 10 nm CPD / 5 nm HATCN / 100 nm Al.

<u>EO devices</u> – ITO (untreated) / 5 nm Alq_3 / 10 nm mCBP / 40 nm EML_x / 10 nm mCBP / 5 nm Alq_3 / 1.5 nm Liq / 100 nm Al.

Here, HO and EO devices have the same EML structures (denoted as EML_1 – EML_4) with the blue dopant [$Ir(dmp)_3$] and the manager [*mer*-$Ir(pmp)_3$] doped at various concentrations into the host (mCBP). Specifically:

EML_1 – mCBP : $Ir(dmp)_3$, 85 : 15 vol%

EML_2 – mCBP : $Ir(dmp)_3$: *mer*-$Ir(pmp)_3$, 85 : 10 : 5 vol%

EML_3 – mCBP : $Ir(dmp)_3$: *mer*-$Ir(pmp)_3$, 80 : 15 : 5 vol%

EML_4 – mCBP : $Ir(dmp)_3$: *mer*-$Ir(pmp)_3$, 75 : 15 : 10 vol%

Supplementary Figure 2 shows the current density (J)–voltage (V) characteristics of HO and EO devices with different EML configurations. Comparing the J–V characteristics of HO with EML_1 vs. EML_2, the latter containing the manager with the reduced blue dopant concentration has the lower conductivity. This indicates hole injection and transport by the manager is less efficient than by the blue dopant, presumably due to the deeper highest occupied molecular orbital (HOMO) level of the manager[2] (5.3 ± 0.1 vs. 4.8 ± 0.1 eV for the blue dopant). As the concentration of the manager is increased (i.e. 5 and 10 vol% for EML_3 and EML_4, respectively), the HO device becomes more conductive at $V > 6$ V, indicating that the manager can facilitate the hole transport. Thus, we can infer that holes are transported by both the blue dopant and the manager from the J-V characteristics of the HO devices.

From the EO devices with a 15 vol% concentration of the blue dopant, the conductivity is nearly independent of both the manager and host concentration (see EML_1, EML_2, and EML_4). Thus, electrons are transported by both the host and manager presumably due to their identical lowest unoccupied molecular orbital energies of 1.5 ± 0.1 eV[3].

This measurement indicates that the manager conducts both electrons and holes which can lead to direct exciton formation on the manager. However, the lack of manager emission provides evidence of rapid energy transfer of $T_M \rightarrow T_1$ as shown in **Fig. 1a** of the main text.

Supplementary Note 3 | Local triplet exciton density profile of the EML

Supplementary Figure 3 shows the local triplet exciton density profile, $N(x)$, of the EML of GRAD PHOLED (see text) at current densities of $J = 0.1$, 1, 5, 10 and 100 mA/cm^2. At $J < 0.1$ mA/cm^2, excitons are formed close to the cathode side of the EML, indicating more efficient hole penetration and transport than that of electrons. This is due to the LUMO energy barrier of ~0.5 eV between the electron transport layer (Alq_3) and the hole blocking layer (mCBP). At higher J or high electric field, the electron density exceeds that of holes in the EML, shifting the exciton profile towards the anode[4]. Note that $N(x)$ at the interface between hole transport layer (HTL) and EML at $x = 0$ is nonzero, as opposed to the case where the EML is in direct contact with the hole injection layer, HATCN[1], which quenches excitons. However, $N(x)$ drops substantially near $x \sim 0$ nm, indicating that the triplet energy of the HTL is insufficient to prevent excitons from leaking from the EML. Nevertheless, the fluorescence from the HTL is absent, indicative of efficient electron blocking by the HTL.

The operating voltages of sensing devices with the ultrathin or "delta-doped" sensing layer (i.e. ~1Å-thick neat PQIr) at different locations are independent of position and also is independent of the presence of the sensing layer itself (**Supplementary**

Figure 3). This indicates that the sensing layer does not significantly affect charge transport in the EML, confirming the validity of the measured exciton profile.

Supplementary Figure 4 shows the simulated power distributions of sensor layer emission in the PHOLED EML as a function of its distance from the mCBP HBL/EML interface using Green's function analysis[5]. The percentage of the power extracted from the device into air, is denoted as Outcoupling in **Supplementary Figure 4**, and corresponds to the outcoupling efficiency, η_{out}, in **Eq. (2)**.

Supplementary Note 4 | Thermally stimulated current

To identify the change in energy distribution and density of the trap states as the PHOLED ages, we measured the thermally stimulated current (TSC) characteristics for GRAD and M3 PHOLEDs. The encapsulated PHOLED was placed in the cryostat at a base pressure of < 2×10^{-2} Torr and was cooled to an initial temperature of T_i = 80 K. After the temperature of the device is stabilized for 15 min, the devices are forward biased to obtain J = 2 mA/cm^2 for 5 min to charge traps in the device. Then, the temperature is increased from T_i to the final temperature of T_f = 340 K at a rate of 5 K/min.

During the temperature ramp, a constant reverse bias at V = –50 meV is applied to the device to extract thermally detrapped charges. A Keysight 4156C precision semiconductor parameter analyzer was used to bias the sample and measure the TSC with a resolution of ~1 fA.

Supplementary Figure 5 shows the TSC of GRAD and M3 managed PHOLEDs measured at T100 and T80. Both devices show high TSC at $T < 100$ K and $T > 300$ K, resulting from de-trapped charges from the transport levels. At $T < 100$ K, the TSC decreases for both devices as they age to T80, indicating that the density of transport sites is depleted. However, the reduction in TSC signal for M3 in this temperature regime is smaller than that for GRAD, yielding a change in charge density of $n_{\mathrm{TSC}} = -(0.7\pm0.1)\times10^{16}$ cm^{-3} vs. $-(1.1\pm0.1)\times10^{16}$ cm^{-3} for M3 and GRAD, respectively. This suggests that molecular transport sites are more rapidly degraded in GRAD than M3. Interestingly at $T > 300$ K, the TSC *decreases* for M3, yielding $n_{\mathrm{TSC}} = -(1.7\pm0.1)\times10^{16}$ cm^{-3} vs. an *increase* in GRAD by $n_{\mathrm{TSC}} = (1.3\pm0.1)\times10^{16}$ cm^{-3}. This is attributed to the increased density of the deep trap states for GRAD which overwhelms the decrease in density of the transport sites, whereas this trend is not observed for M3. A broad peak in the TSC appears for the degraded M3 between 125 K $< T <$ 280 K with larger $n_{\mathrm{TSC}} = (4.7\pm0.1)\times10^{16}$ cm^{-3} compared to that for GRAD with $n_{\mathrm{TSC}} = (2.1\pm0.1)\times10^{16}$ cm^{-3}. This suggests that a species of shallow defects is generated in M3 that is not present in GRAD after aging. These additional shallow traps increase the trapped polaron density in the transport layers of the managed PHOLEDs, which is consistent with the lifetime model and the faster voltage rise of the managed devices. For example, assuming that the energetic distribution of external defects (Q_{ext}) is uniform, the densities of shallow defects calculated from the lifetime model are $(2.9\pm0.1)\times10^{16}$ cm^{-3} and $(4.3\pm0.1)\times10^{16}$ cm^{-3} for GRAD and M3 at their T80, respectively, consistent with their n_{TSC} values.

Thus, although the TSC is limited in its ability to precisely determine the energy levels and location of the traps, we found (i) decreased density of transport sites and (ii) a

growing density of defects as devices age, as well as (iii) distinctive features of the managed PHOLEDs compared to GRAD that are attributed to the protected EML by the manager, and new signals generated from degradation products of the manager.

Supplementary Note 5 | Sensitivity test of the lifetime model

Supplementary Figure 6 shows the contributions of the various defects to the fits. The lifetime data, i.e. time evolution of the luminance degradation and the voltage rise, of the managed PHOLED M1 is used as an example.

Compared to the model with a complete set of defects (black solid line, see text), the one excluding Q_{ext} (red dashed line) underestimates $\Delta V(t)$, while providing a reasonable fit for $L(t)/L_0$. This is because $L(t)/L_0$ is affected only by the defects *within* the EML ($Q_{EML} = Q_A + Q_B$), as well as the electrons, holes, and excitons whose densities are determined by the constant current density of J_0 and the measured recombination profile of $G(x) = J_0 \cdot N(x) \big/ e \cdot \int_{EML} N(x)dx$. On the other hand, the voltage rise due to defects Q_A and Q_B within the EML [$\Delta V_{EML}(t)$] is insufficient to account for the measured $\Delta V(t)$ (see **Fig. 5c** in text). Thus the defects present outside of the EML [$\Delta V_{ext}(t)$] need to be included.

If only one type of EML defect (Q_A) is assumed to be active along with Q_{ext}, the fit for both $L(t)/L_0$ and $\Delta V(t)$ significantly deviates from the data. This necessitates the inclusion of shallow defects (Q_B) that trap charges but also contribute to the PHOLED luminescence via transfer back to the dopant molecule (see text). Finally, the fit from the

model[7] assuming only Q_A and triplet-polaron annihilation (TPA) for the defect generation is also shown to deviate from data.

Supplementary Figure 7 shows the sensitivity to variations of a single fitting parameter used in the lifetime model. From the calculated set of parameters (yielding the black solid line, denoted as "Best"), each parameter is modified by an order of magnitude within the bounds for the fit, while the other parameters are fixed. The results show that the changes in k_{QN}, k_{QA}, k_{QB} and k_{QP} result in significant deviations from the measurements. k_{Qext} does not affect $L(t)/L_0$ whereas it strongly influences $\Delta V(t)$, consistent with the data shown in **Supplementary Figure 6**.

Note that the initial values (I.V.) chosen for the fitting parameters are set within the upper and lower bounds that are an order of magnitude larger or smaller, respectively, relative to the final parameters providing the best fit (see **Table 2** in text). The fitting parameters with arbitrary I.V. within such boundary conditions (B.C.) converge to the final values reported, while if I.V. of one or more parameters are set outside the given B.C. lead to different values with an unacceptably poor fit to the data.

We also forced a single parameter to be smaller or larger by an order of magnitude relative to its final value, and the induced discrepancy in the fit was attempted to be compensated by varying the remaining four parameters. **Supplementary Figure 8** shows that all the resulting simulations cannot fit the voltage rise, while some of those with changed k_{QN}, k_{Qp} and k_{Qext} are in a reasonable agreement with the luminance degradation. However, considering that these fitting parameters are derived from coupled equations that should predict *both* the lifetime and voltage characteristics, the models that satisfy only one of these two characteristics are not accepted.

Supplementary References

1. Zhang, Y., Lee, J. & Forrest, S. R. Tenfold increase in the lifetime of blue phosphorescent organic light-emitting diodes. Nat. Commun. 5, (2014).

2. Lee, J. et al. Deep blue phosphorescent organic light-emitting diodes with very high brightness and efficiency. Nat. Mater. 15, 92–98 (2016).

3. Djurovich, P. I., Mayo, E. I., Forrest, S. R. & Thompson, M. E. Measurement of the lowest unoccupied molecular orbital energies of molecular organic semiconductors. Org. Electron. 10, 515–520 (2009).

4. Coburn, C., Lee, J. & Forrest, S. R. Charge Balance and Exciton Confinement in Phosphorescent Organic Light Emitting Diodes. Adv. Opt. Mater. 4, 889–895 (2016).

5. Celebi, K., Heidel, T. D. & Baldo, M. A. Simplified calculation of dipole energy transport in a multilayer stack using dyadic Green's functions. Opt. Express 15, 1762–1772 (2007).

6. Sajoto, T. et al. Blue and Near-UV Phosphorescence from Iridium Complexes with Cyclometalated Pyrazolyl or N-Heterocyclic Carbene Ligands. Inorg. Chem. 44, 7992–8003 (2005).

7. Giebink, N. C. et al. Intrinsic luminance loss in phosphorescent small-molecule organic light emitting devices due to bimolecular annihilation reactions. J. Appl. Phys. 103, 44509 (2008).

8. Oh, C. S., Choi, J. M. & Lee, J. Y. Chemical Bond Stabilization and Exciton Management by CN Modified Host Material for Improved Efficiency and Lifetime in Blue Phosphorescent Organic Light-Emitting Diodes. Adv. Opt. Mater. 4, 1281–1287 (2016).

9. Kang, Y. J. & Lee, J. Y. High triplet energy electron transport type exciton blocking materials for stable blue phosphorescent organic light-emitting diodes. Org. Electron. 32, 109–114 (2016).

10. Zhang, L. et al. Highly Efficient Blue Phosphorescent Organic Light-Emitting Diodes Employing a Host Material with Small Bandgap. ACS Appl. Mater. Interfaces 8, 16186–16191 (2016).

11. Seo, J.-A. et al. Long lifetime blue phosphorescent organic light-emitting diodes with an exciton blocking layer. J. Mater. Chem. C 3, 4640–4645 (2015).

12. Lee, S. E. et al. Optimization of hybrid blue organic light-emitting diodes based on singlet and triplet exciton diffusion length. Jpn. J. Appl. Phys. 54, 06FG09 (2015).

13. Zhuang, J. et al. Highly efficient phosphorescent organic light-emitting diodes using a homoleptic iridium(III) complex as a sky-blue dopant. Org. Electron. 14, 2596–2601 (2013).

14. Klubek, K. P., Dong, S.-C., Liao, L.-S., Tang, C. W. & Rothberg, L. J. Investigating blue phosphorescent iridium cyclometalated dopant with phenyl-imidazole ligands. Org. Electron. 15, 3127–3136 (2014).

15. Fleetham, T. B., Huang, L., Klimes, K., Brooks, J. & Li, J. Tetradentate Pt(II) Complexes with 6-Membered Chelate Rings: A New Route for Stable and Efficient Blue Organic Light Emitting Diodes. Chem. Mater. 28, 3276–3282 (2016).

16. Jeon, S. K. & Lee, J. Y. Four times lifetime improvement of blue phosphorescent organic light-emitting diodes by managing recombination zone. Org. Electron. 27, 202–206 (2015).

17. Yamamoto, H. et al. Improved initial drop in operational lifetime of blue phosphorescent organic light emitting device fabricated under ultra high vacuum condition. Appl. Phys. Lett. 99, 33301 (2011).
